# Malignant Epithelioid Neoplasm of the Brain

**DOI:** 10.7759/cureus.10079

**Published:** 2020-08-27

**Authors:** Abhishrut P Jog, Diana M Ronderos, Asghar Ali, Masooma Niazi, Gilda Diaz-Fuentes

**Affiliations:** 1 Internal Medicine, BronxCare Health System, Bronx, USA; 2 Pulmonology and Critical Care, BronxCare Health System, Bronx, USA; 3 Pathology, BronxCare Health System, Bronx, USA; 4 Pulmonary and Critical Care, BronxCare Health System, Bronx, USA

**Keywords:** primary brain tumors

## Abstract

Malignant epithelioid tumors have been described in various organ systems, but are rarely seen in the brain. They are aggressive tumors and have high mortality. In certain cases, the immunohistochemistry (IHC) findings may not be sufficient to clarify the diagnosis. In these cases, next-generation genetic sequencing may play a role in clarifying the diagnosis. In addition to lab testing, a thorough history and physical exam are necessary to rule out other sources of the tumor such as melanoma. Patients presenting with neurological symptoms are cared for by a wide variety of physicians, hence it is important to raise awareness of rare tumors in order to provide timely and appropriate management and referral for these patients. We present the case of a middle-aged woman who was diagnosed with a ‘malignant epithelioid neoplasm’ of the brain, a rare variety of tumors. We also give the clinical course of this illness.

## Introduction

In the United States, the annual incidence of all tumors involving the brain is 46 per 100,000, and that of primary brain tumors is 15 per 100,000. Out of the total 600,000 cancer-related deaths that occur annually in the country, around 20,000 are attributable to primary brain tumors. The most common types of primary intracranial neoplasms are meningioma, pituitary tumors, and gliomas [1]. Epithelioid neoplasms are not a very well described entity of brain tumors. Literature review reveals descriptions of epithelioid neoplasms in a variety of organ systems, but only a few in the brain, with further paucity of data on malignant forms.

We present the case of a middle-aged woman who was diagnosed with a ‘malignant epithelioid neoplasm’ and give the clinical course, diagnosis, and outcome of this illness.

## Case presentation

A 57-year-old female with a history of hypertension and HIV, compliant with highly active antiretroviral therapy (HAART) (CD4- 392 with undetectable viral load) presented with sudden onset left-sided weakness. Clinical exam revealed a power of 2/5 in the left upper and lower extremities, along with facial deviation to the right side. A thorough history and physical exam (head to toe, including back, palms, soles, and anal region) was found to be negative as well. The patient had no history of a similar episode or of any tumors in the past. There was no history of exposure to ionizing radiation or a known carcinogen in the past. Additionally, the patient did not have a history of any familial ailment predisposing to cancer.

**Figure 1:**
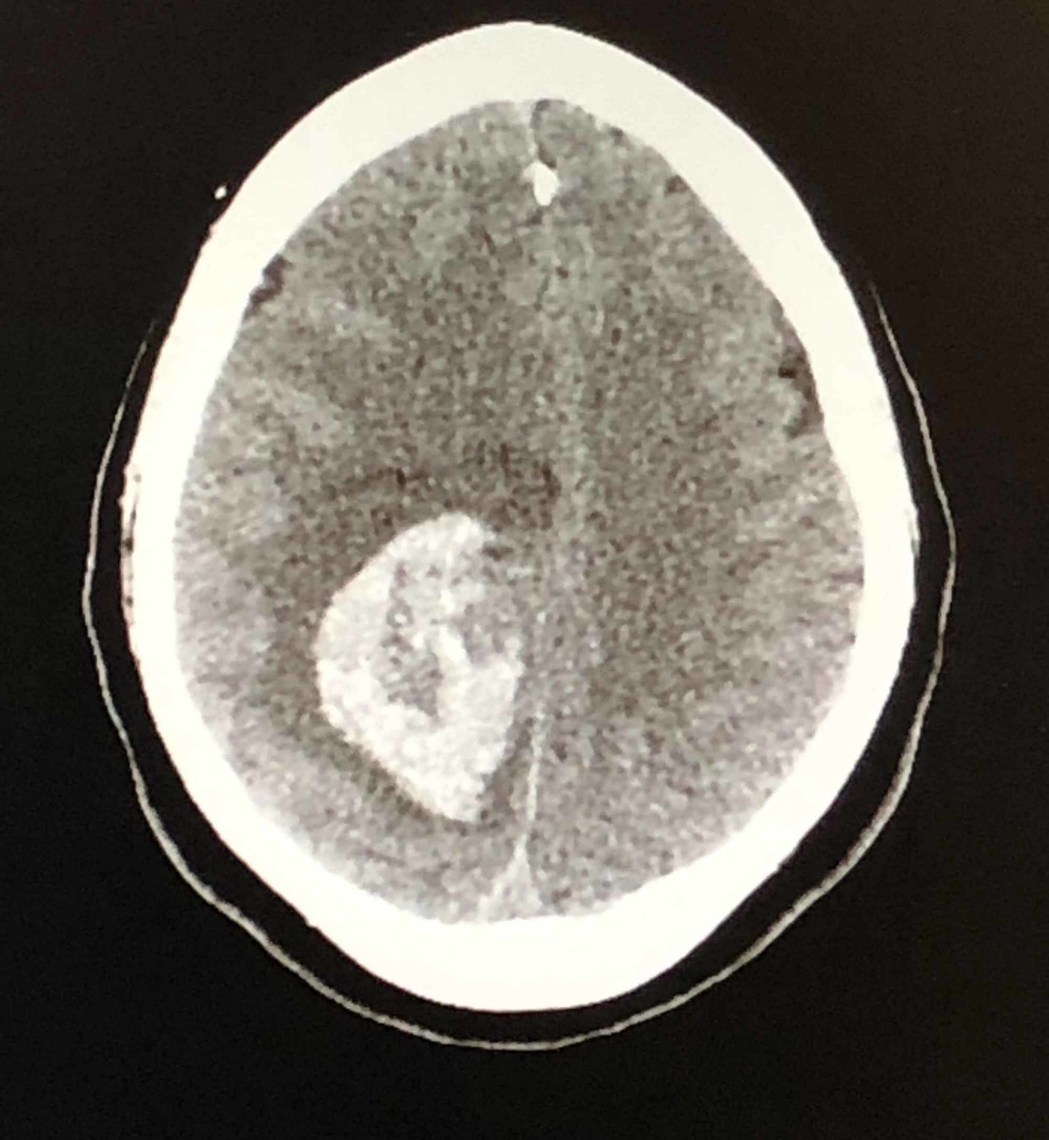
Non-contrast CT head Large acute hemorrhage in the right centrum semiovale measuring approximately 5.7 x 3.8 x 3.8 cm resulting in inferior displacement and effacement of the right lateral ventricle and approximately 8 mm of right-to-left midline shift.

Non-contrast CT head found a large hemorrhagic stroke measuring 5.7 x 3.8 x 3.8 cm in the right centrum semiovale resulting in inferior displacement and effacement of the right lateral ventricle and approximately 8 mm of right-to-left midline shift (Figure 1). A right parietooccipital craniotomy and removal of an intracerebral clot were performed.

**Figure 2:**
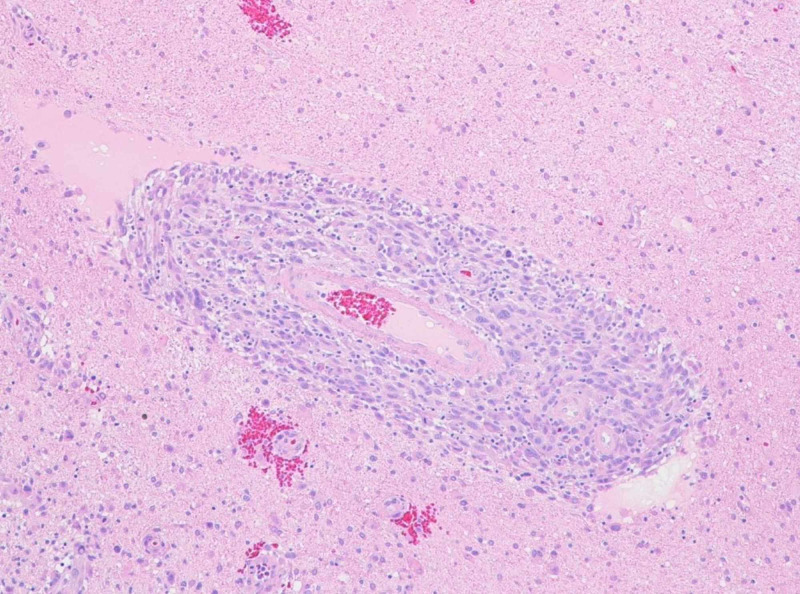
Light microscopy showing perivascular arrangement of tumor cells In the perivascular areas, the tumor cells show a concentric growth pattern around blood vessels (H&E, magnification x 200). H&E, hemotoxylin and eosin.

**Figure 3:**
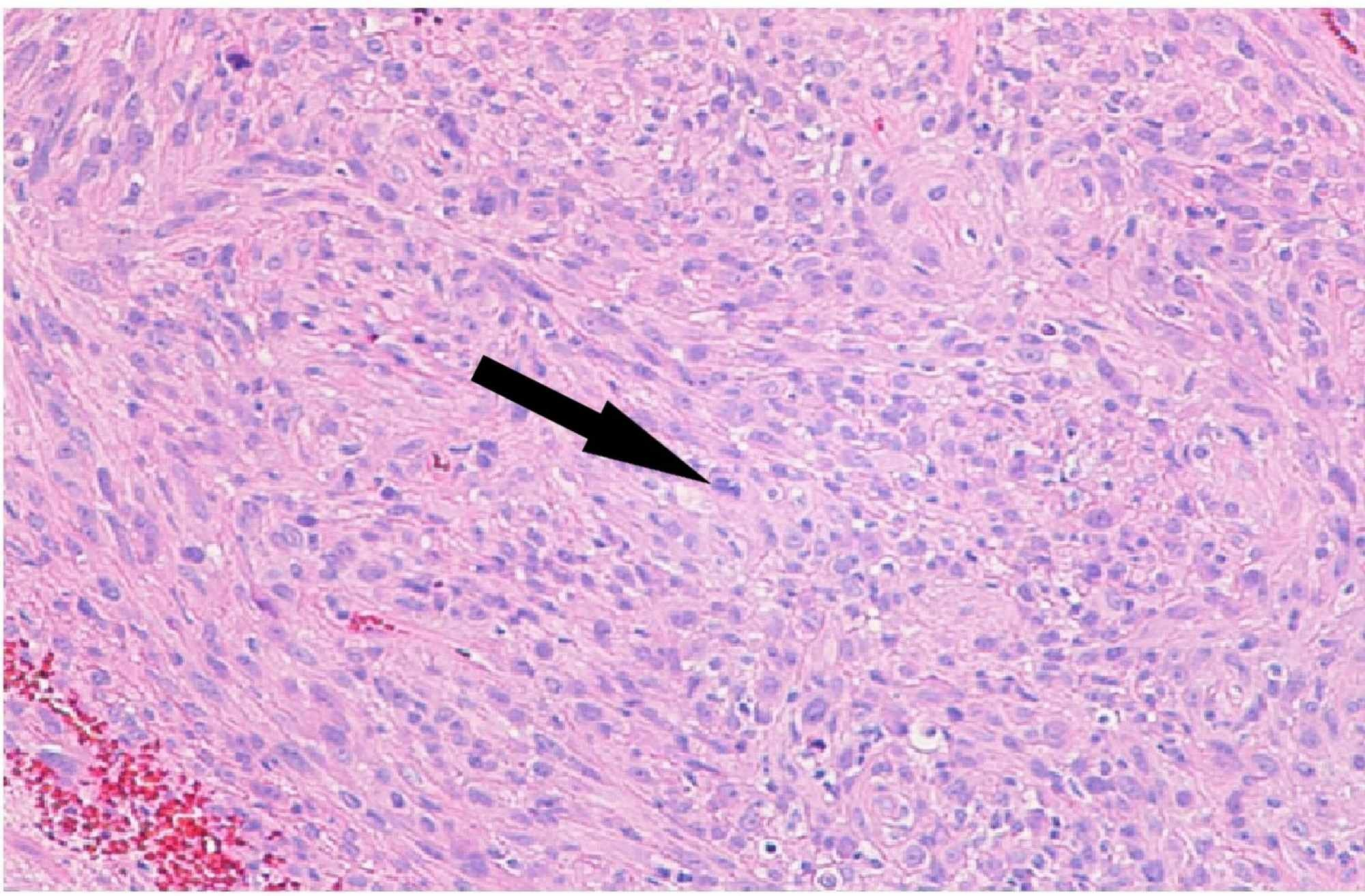
Light microscopy showing epithelioid cells The tumor is comprised of predominantly epithelioid cells and focally spindle cells growing in sheets and fascicles. The cells are pleomorphic with eosinophilic cytoplasm and multiple mitotic figures (arrow). H&E magnification x200. H&E, hemotoxylin and eosin.

Grossly the tissue was light tan in color and soft in consistency. Microscopic examination of hematoxylin and eosin-stained sections of the specimen showed an epithelioid and focally spindle-cell neoplasm that was mainly distributed in the perivascular Virchow-Robin spaces and in the leptomeningeal spaces (Figures 2, 3). The tumor was found to grow in sheets and fascicles. In the perivascular areas, the tumor showed a concentric growth pattern around blood vessels. Focally, there was a myxoid matrix deposition. The neoplastic cells were epithelioid and markedly pleomorphic, with a round to spindle-shaped, often irregular, hyperchromatic nuclei and occasionally prominent eosinophilic nucleoli. The neoplastic cells had moderate amounts of eosinophilic, sometimes granular, cytoplasm and showed distinct cell boundaries in some areas, but appeared syncytial in others. Multinucleated cells were also seen focally. Multiple mitotic figures were noted (including atypical mitoses). The surrounding brain parenchyma showed reactive astrocytosis, foamy macrophages, edema, and perivascular inflammation. CD45 highlighted many tumor-infiltrating immune cells and CD68 highlighted many tumor-infiltrating macrophages. The Ki 67 proliferation index was elevated, with up to 20% of cells labeling positive.

**Figure 4:**
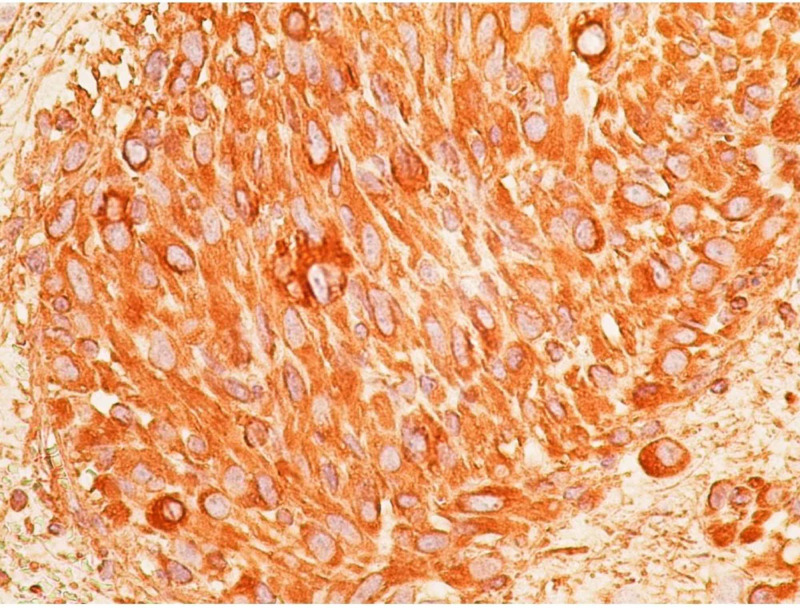
Immunoreactivity to vimentin The tumor cells are strongly immunoreactive to vimentin (immunohistochemistry, magnification x 400).

On IHC, the following markers were found to be positive in our patient: vimentin, caldesmon, Melan-A, Human Melanoma Black (HMB)-45, S 100, SOX 2, oligodendrocyte transcription factor 2 (Olig 2), platelet-derived growth factor receptor (PDGFR) alpha, cluster of differentiation (CD) 34, integrase interactor 1 (INI-1) (preserved) (Figure 4).

The following markers were found to be negative in our patient: GFAP, CD 31, cytokeratin (CK), CK CAM 5.2, p40, p63, ETS-related gene (ERG), Desmin, spinal muscular atrophy (SMA), smooth muscle myosin (SMMS-1), calponin, myogenin, myoblast determination protein 1 (Myo D1), microphthalmia-associated transcription factor (MITF), tyrosinase, SOX 10, c-kit, signal transducer and activator of transcription (STAT 6), PR, ER, CD 10, Epstein-Barr virus (EBV), human herpesvirus (HHV)-8.

The findings did not conform to a specific diagnosis but were suggestive of an entity called a PEComa (Perivascular Epithelioid Cell neoplasm). A diagnosis of a ‘malignant epithelioid neoplasm’ was made.

A CT abdomen-pelvis and CT chest were done to look for a possible primary source but were found to be negative. MRI brain following the resection showed post-operative changes and mild fluid collection.

Despite the initial improvement, the patient complained of gradually worsening dizziness over the next four months. A repeat MRI showed a large hemorrhage in the cingulate gyrus bilaterally just above the posterior third of the body of the corpus callosum. Mass effect compression of the corpus callosum and a large amount of vasogenic edema extending into both parietal lobe white matter was present. Following the injection of gadolinium contrast, prominent enhancement within the region of the hemorrhage was seen. This was strongly suspicious of hemorrhagic neoplasm.

**Figure 5:**
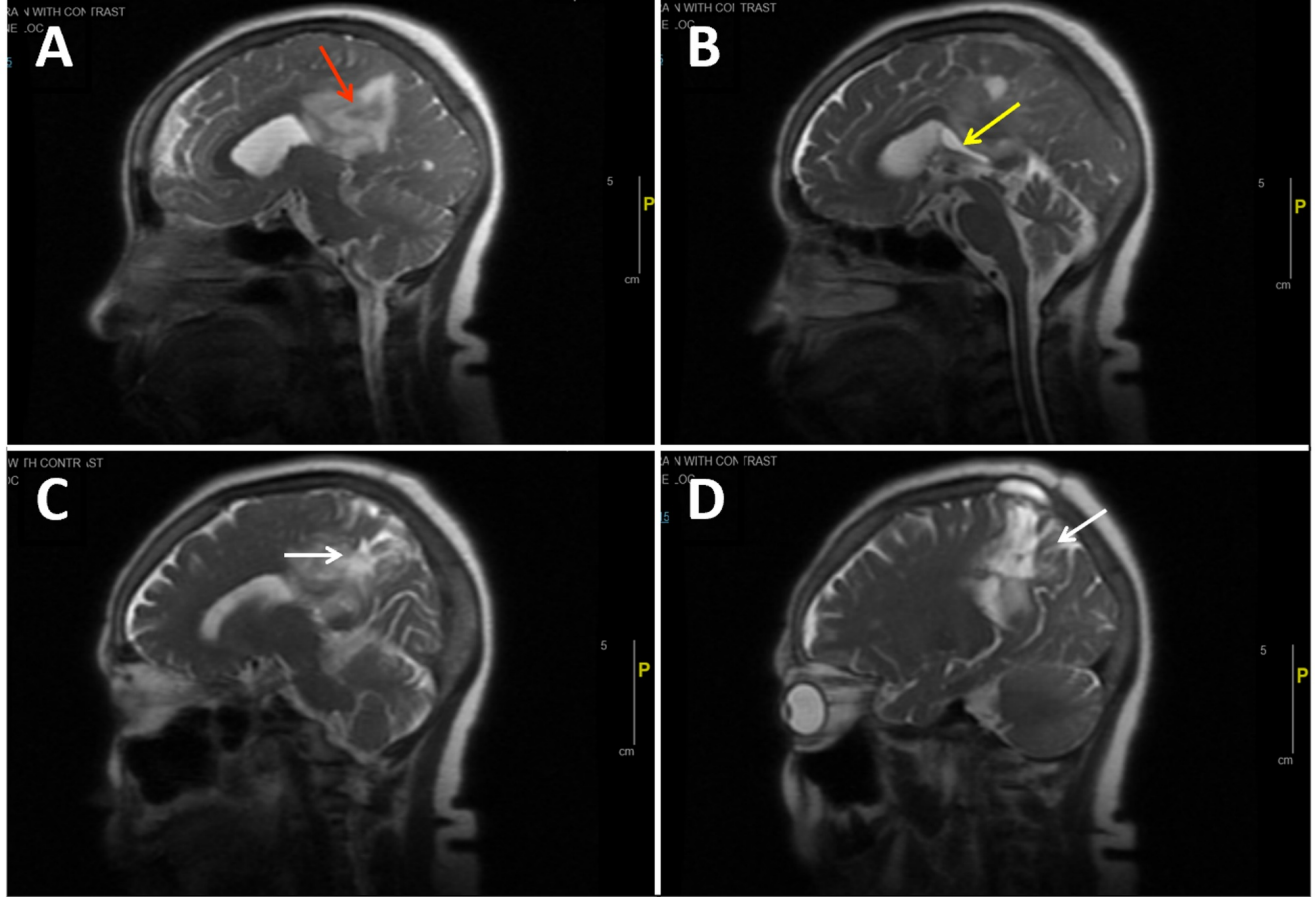
Magnetic resonance imaging of the brain A: large hemorrhage in the cingulate gyrus (red arrow); B: mass effect compresses the corpus callosum (yellow arrow); C and D: a large amount of vasogenic edema extends into both parietal lobe white matter (white arrows).

A stereotactic biopsy was performed. The tissue showed only reactive gliosis. The patient did not opt for further treatment. She was re-admitted three months later due to a fall. MRI brain on this admission showed an enhancing mass involving the posterior aspect of the corpus callosum extending to both parietal lobes measuring 5.1 x 3.9 x 4.3 cm, a progression from the prior MRI (Figure 5). Additionally, a bilateral pulmonary embolism was found. The hospital course was also complicated by status epilepticus. The patient’s condition deteriorated within the next month and she succumbed to the illness.

## Discussion

Immunohistochemical (IHC) staining was helpful in sifting through various tumor differentials that arose in this case. Glioblastoma, metastatic melanoma, angiosarcoma, epithelioid sarcoma, sarcomatoid carcinoma, meningeal hemangiopericytoma, and paraganglioma were all considerations. Glioblastomas typically show GFAP positivity [2]. In this case, glial fibrillary acidic protein (GFAP) was negative. For melanocytic tumors, S 100 is the most sensitive marker. HMB-45, Melan-A, tyrosinase, and MITF demonstrate good specificity but not as good sensitivity as S-100 in melanomas [3]. In our case, S 100, HMB-45, and Melan A were positive, whereas tyrosinase and MITF were negative. Additionally, more recently, Willis et al showed the usefulness of the marker SOX 10 in identifying metastatic melanomas [4]. There was a 100% sensitivity and specificity in diagnosing melanomas. In our case, SOX 10 was negative. A melanoma had also been ruled out clinically. ERG and CD31 negativity went against angiosarcoma [5]. The preserved INI-1 goes against epithelioid sarcoma [6]. Sarcomatoid carcinomas typically show positivity for CK [7], which was not the case in our patient. Absence of STAT- 6 goes against a diagnosis of meningeal hemangiopericytoma [8]. Chromogranin and synaptophysin studies which are usually positive in paragangliomas were not available; however, imaging was not suggestive of that [9].

The tumor that was characterized as a ‘malignant epithelioid neoplasm’ showed some features of the tumor entity called PEComa. The World Health Organization (WHO) defines PEComa as: ‘mesenchymal tumors composed of histologically and immunohistochemically distinctive perivascular epithelioid cells (PECs)' [10]. Microscopically, the perivascular epithelioid cells are characterized by a radial arrangement around a blood vessel as seen in this case. They may show the presence of two components: an immediate perivascular component that appears epithelioid and a spindle shape appearing component, away from the vessel. They have a clear to granular, lightly eosinophilic cytoplasm. The nuclei are usually round to oval, centrally placed, and normochromic and may display mild to a significant amount of nuclear atypia and mitotic activity [11]. IHC in PEComa’s classically shows strong positivity for melanocytic markers, HMB-45 and Melan A, and myogenic markers such as SMA, desmin, Myo D1 [12]. In this case, HMB-45 and Melan A were weakly positive. Caldesmon was the myogenic marker that was positive, whereas the other myoid markers were absent. Even though the tumor does not fit all the characteristics of a PEComa, an atypical presentation of this rare tumor is a possibility. No other source of malignancy was found in this case, despite an extensive search.

In our case, a few points are suggestive of the malignant potential: tumor size > 5 cm, the Ki 67 index is 20%, multiple mitoses including atypical mitotic bodies are present, recurrent growth of the tumor with hemorrhagic transformation and rapid and fatal course of the illness.

Treatment decisions for intracranial neoplasms are individualized and need a multidisciplinary approach consisting of medical oncology, radiation oncology, and neurosurgery. They are based on tumor type, location, malignancy potential, patient’s age, and physical condition. Treatment commonly includes surgery, radiotherapy, chemotherapy, or a combination [13]. Treatment for most PEComa’s has been through surgical excision. The recognition of PEComa has important management implications as some of them respond to mammalian target of rapamycin (mTOR) inhibitors such as sirolimus or everolimus [14]. In our case, the patient had a surgical resection resulting in a temporary decrease of symptoms; however, the tumor grew back within the next four months.

The patient was HIV-positive. The most common primary tumor in HIV-positive individuals is a primary central nervous system (CNS) lymphoma. The occurrence of this malignant epithelioid neoplasm in the patient may be unrelated to the viral infection, and an association between the two has not been described in the literature. However, it is worth further investigation. The patient had multiple risk factors for the development of a pulmonary embolism including immobility due to the stroke, tumor, and surgery in the form of craniotomy. Greater clarity about the tumor can be obtained through next-generation sequencing, which was not done in this case.

## Conclusions

Malignant epithelioid tumors are uncommon tumors in the brain. They are aggressive tumors and have high mortality. In certain cases, the IHC findings may not be sufficient to clarify the diagnosis. In these cases, next-generation genetic sequencing may play a role in clarifying the diagnosis. In addition to lab testing, a thorough history and physical exam are necessary to rule out other sources of the tumor such as melanoma. Patients presenting with neurological symptoms are cared for by a wide variety of physicians, hence it is important to raise awareness of rare tumors in order to provide timely and appropriate management and referral for these patients.
